# Potential Diagnostic, Prognostic and Therapeutic Targets of MicroRNAs in Human Gastric Cancer

**DOI:** 10.3390/ijms17060945

**Published:** 2016-06-16

**Authors:** Ming-Ming Tsai, Chia-Siu Wang, Chung-Ying Tsai, Hsiang-Wei Huang, Hsiang-Cheng Chi, Yang-Hsiang Lin, Pei-Hsuan Lu, Kwang-Huei Lin

**Affiliations:** 1 Department of Nursing, Chang-Gung University of Science and Technology, Taoyuan 333, Taiwan; mmtsai@mail.cgust.edu.tw; 2 Department of General Surgery, Chang Gung Memorial Hospital, Chiayi 613, Taiwan; wangcs@adm.cgmh.org.tw; 3 Department of Biochemistry, College of Medicine, Chang-Gung University, Taoyuan 333, Taiwan; monster_0616@yahoo.com.tw (C.-Y.T.); memorywind@hotmail.com (H.-W.H.); d9501111@stmail.cgu.edu.tw (H.-C.C.); sam4915@yahoo.com.tw (Y.-H.L.); 4 Department of Dermatology, Chang Gung Memorial Hospital and Chang Gung University College of Medicine, Taoyuan 333, Taiwan; peihsuanlu0613@gmail.com; 5 Liver Research Center, Chang Gung Memorial Hospital, Linkou, Taoyuan 333, Taiwan

**Keywords:** biomarker, prognosis, gastric cancer, diagnosis, microRNAs

## Abstract

Human gastric cancer (GC) is characterized by a high incidence and mortality rate, largely because it is normally not identified until a relatively advanced stage owing to a lack of early diagnostic biomarkers. Gastroscopy with biopsy is the routine method for screening, and gastrectomy is the major therapeutic strategy for GC. However, in more than 30% of GC surgical patients, cancer has progressed too far for effective medical resection. Thus, useful biomarkers for early screening or detection of GC are essential for improving patients’ survival rate. MicroRNAs (miRNAs) play an important role in tumorigenesis. They contribute to gastric carcinogenesis by altering the expression of oncogenes and tumor suppressors. Because of their stability in tissues, serum/plasma and other body fluids, miRNAs have been suggested as novel tumor biomarkers with suitable clinical potential. Recently, aberrantly expressed miRNAs have been identified and tested for clinical application in the management of GC. Aberrant miRNA expression profiles determined with miRNA microarrays, quantitative reverse transcription-polymerase chain reaction and next-generation sequencing approaches could be used to establish sample specificity and to identify tumor type. Here, we provide an up-to-date summary of tissue-based GC-associated miRNAs, describing their involvement and that of their downstream targets in tumorigenic and biological processes. We examine correlations among significant clinical parameters and prognostic indicators, and discuss recurrence monitoring and therapeutic options in GC. We also review plasma/serum-based, GC-associated, circulating miRNAs and their clinical applications, focusing especially on early diagnosis. By providing insights into the mechanisms of miRNA-related tumor progression, this review will hopefully aid in the identification of novel potential therapeutic targets.

## 1. Introduction

Gastric cancer (GC), a malignant epithelial cancer disease [1], is associated with a high global incidence of mortality [2,3]. Although surgical resection, together with chemotherapy and radical therapy, shows significant improvement over surgery alone in early-stage GC patients [4,5], GC patients commonly present with late-stage cancer at initial diagnosis owing to the lack of clinical symptoms that would enable early detection [2,3,6]. The five-year survival rate for late-stage GC patients is only about 20%–30% [7]. Thus, additional studies designed to improve early detection of GC are needed to provide better quality of life and longer survival for GC patients. Early diagnosis is critical for greatly reducing the efficiency of peritoneal spread and local/distal metastasis of GC, necessitating the development of new and more sensitive tumor markers for early GC diagnosis and disease monitoring. Conventional plasma/serum-based tumor biomarkers commonly used clinically for early GC diagnosis, including carcinoembryonic antigen (CEA), the carbohydrate antigens (CA), CA19-9, CA72-4, CA125, CA24-2 and CA50, as well as pepsinogen and α-fetoprotein (AFP), have poor specificity and sensitivity [8,9].

MicroRNAs (miRNAs) are small (~22 bp) nucleic acids that function by regulating the expression of downstream target genes [10]. Their dysregulation has been reported to be involved in pathogenic processes underlying GC tumorigenesis and progression, including cell growth, invasion, metastasis, and apoptosis. Moreover, miRNAs are stable and persistent among individuals of the same species, even for several years in formalin-fixed, paraffin-embedded tissues and body fluids, such as plasma/serum, urine, saliva, and milk [11,12,13,14,15]. Therefore, aberrantly expressed miRNAs are potentially useful biomarkers for GC screening, diagnosis, prognosis and disease monitoring, as well as therapeutic targets.

A number of researchers have explored the possibility of using miRNAs as biomarkers. Here, we summarize major, up-to-date information on the subject, focusing on discoveries from systematic analysis of miRNA profiling, microarray profiling and quantitative reverse transcription-polymerase chain reaction (Q-RT-PCR) profiling approaches. Specifically, we discuss plasma/serum-based, GC-related circulating miRNAs and their clinical application, focusing particularly on their application as diagnostic and prognostic indicators. We also review tissue-based, GC-related miRNA biomarkers and their downstream targets in GC, as well as plasma/serum-based, GC-associated circulating miRNAs and their clinical applications, focusing especially on early diagnosis. Moreover, we examine correlations among significant clinical parameters and prognostic indicators, and discuss recurrence monitoring and therapeutic options in GC. miRNA biomarkers with potential applications in GC are listed in Table 1, Table 2, Table 3 and Table 4.

In order to search of all the related literatures, we used PubMed for the GC microRNA expression profiling studies between January 2000 and December 2016. The keyword “miR and gastric cancer” was used. Selected studies should fit the following search criteria: (1) profiling studies in GC patients; (2) including the appropriate adjacent noncancerous gastric tissues or normal plasma/serum for control; (3) including the known cut-off criteria/value of differentially expressed miRNAs; and (4) including the known number of study patients or normal subjects; (5) showing statistical analysis data.

## 2. Cellular Functions of miRNAs in GC

Aberrantly expressed miRNAs serve oncogenic or tumor-suppressor functions in tumorigenesis. They can regulate cell proliferation, cell cycle progression, apoptosis, angiogenesis, cell migration, cell invasion and/or metastasis in GC (Table 1 and Table 2), depending on their target genes. Therefore, a given miRNA may exert dual, opposite functions in GC. Generally, oncogenic miRNAs (oncomiRs) are over-expressed in GC and act to inhibit tumor-suppressor genes. Conversely, tumor-suppressor miRNAs, which inhibit oncogene expression, are usually down-regulated in GC. miRNAs in this category regulate various biological processes to stimulate cancer development.

### 2.1. GC-Related miRNAs in Cell Proliferation, Cell Cycle, and Apoptosis

Accelerated cell proliferation, cell cycle progression or disturbed apoptosis are common features of malignancy that arise through silencing of cell cycle-inhibitory or apoptotic pathway-associated genes. In several malignant tumors, miRNA dysregulation stimulates cell cycle progression by up-regulating cyclin expression or down-regulating the expression of other cell cycle regulators or cyclin-CDK (cyclin-dependent kinase) inhibitors, including members of the p16 family (p15, p16, p18 and p19) and p21 family (p21, p27, p28 and p57) [212,213,214]. Moreover, transforming growth factor (TGF)-β1 has been shown to repress GC cell proliferation through transcriptional up-regulation of p21 [108]. In this context, oncomiR-106b and oncomiR-93 are both up-regulated in GC and target the downstream E2F1 (E2F transcription factor 1) and p21 (cyclin-dependent kinase inhibitor 1A), thereby inhibiting the activity of TGF-β1 [26] and contributing to GC by enhancing cell proliferation.

In addition, these oncomiR clusters are significantly up-regulated in GC. miR-106b-93-25 and miR-222-221 have been reported to inhibit the p21 family CDK inhibitors p57^KIP2^, p21^CIP1^ and p27^KIP1^ [27]. Kim *et al.* showed that over-expression of the miR-222-221 cluster also enhances the growth of GC xenografts in nude mice [27], further reporting that miR-25 targets p57. In addition, both miR-106b and miR-93 down-regulate p21, whereas miR-222 and miR-221 both control p27 and p57. miR-449, which targets cyclin E2 and geminin, among others, and normally promotes senescence and apoptosis, is down-regulated in GC. Consistent with these biological functions, down-regulation of miR-449 in GC promotes G1/S and M/G1 cell cycle progression and cell proliferation [178]. Cui *et al.* [166] reported that the tumor suppressors miR-449 and miR-29a both target p42.3 (suppressor APC domain containing 2) in GC, promoting increased G2/M cell cycle progression and proliferation. In addition to directly targeting CDK inhibitors, miR-24 also modulates anion exhanger-1 (AE1), and thus promotes cell proliferation [164,165]. Moreover, let-7, which targets CDC34, is frequently down-regulated in GC [105].

Some oncomiRs are significantly up-regulated in GC tissues and target downstream tumor-suppressor genes. Zhang *et al.* [68,69,70,71,72] showed that one such oncomiR, miR-21, directly targets the tumor-suppressor gene RECK (reversion-inducing cysteine-rich protein with kazal motifs) and contributes to GC by enhancing cell proliferation and inhibiting apoptosis. Several lines of evidence have revealed that miR-21 also has the ability to stimulate cell invasion and migration. The oncomiR miR-199a was shown to significantly inhibit SMAD4, thereby inhibiting TGF-β1 signaling control over cell proliferation and apoptosis, and promoting anchorage-independent growth in soft agar [39,58,62,63,64,65]. Another oncomiR, miR-23a, was shown to significantly promote GC cell proliferation by silencing its target, the interleukin (IL)-6 receptor (IL6R) [77,78,79].

Conversely, some tumor-suppressor miRs that target downstream oncogenes are significantly down-regulated in GC tissues. Carvalho *et al.* reported that the tumor suppressor miR-101, which targets *EZH2* (enhancer of zeste 2 polycomb repressive complex 2 subunit), *COX-2* (cytochrome c oxidase subunit II), *MCL-1* (myeloid cell leukemia 1) and *FOS* (FBJ osteosarcoma oncogene), has anti-proliferative and anti-metastatic functions in GC [117,118,119,120]. In addition, the tumor suppressor miR-125a, which targets *ERBB2* (erb-b2 receptor tyrosine kinase 2), and miR-129, which targets *CDK6* (cyclin-dependent kinase 6), are also involved in anti-proliferative and pro-apoptotic functions [24,126,127,131]. Similarly, Song *et al.* showed that the tumor suppressor miR-148b, which targets *CCKBR* (cholecystokinin B receptor), is anti-proliferative *in vitro* and anti-tumorigenic *in vivo* [138]. These results suggest that abnormal miRNA expression may increase cell cycle progression through direct or indirect regulation of CDK inhibitors and cell cycle–associated regulators.

In addition, anti-apoptosis is a character of tumorigenesis [16]. miR-106b and miR-93 abrogate TGFβ-induced apoptosis in GC cells by targeting the expression of *BIM*, encoding the pro-apoptotic protein BCL2-like 11, and thereby prevent apoptosis and cause tumor progression [26]. OncomiR-130b in GC cells increases cell viability and anti-apoptosis by targeting TGFβ-induced RUNX3 (runt related transcription factor 3) [37]. Lai *et al.* have also reported that miR-130b suppresses TGFβ-induced BIM expression and apoptosis by targeting RUNX3 in GC cells. Moreover, several oncomiRs, namely miR-15b, miR-16, miR-181b and miR-34, directly target the gene encoding the anti-apoptotic protein Bcl-2, and thus promote apoptosis in GC. The tumor suppressors miR-15b, miR-16 and miR-181b have been shown to inhibit chemotherapeutic drug-induced apoptosis [47,48]. In addition, oncomiR-150 negatively regulates the pro-apoptotic gene *EGR2* (early growth response 2) to accelerate GC growth [215]. Shen *et al.* [35] reported that miR-129-2 targets *SOX4* to induce apoptosis by regulating the relative abundance of pro-apoptotic and anti-apoptotic members of the Bcl-2 family in GC. Bandres *et al.* [94] reported that miR-451 functions as a tumor suppressor by repressing migration inhibitory factor (MIF), thereby activating Bcl-2, EGFR (epidermal growth factor receptor) and the phosphoinositide 3-kinase (PI3K)/Akt pathway in GC [95,96]. Another study showed that ectopic expression of the tumor suppressor miR-375 reduced cell viability in GC cells through the proliferative PI3K/Akt pathway (by targeting JAK2 and PDK1) and the anti-apoptotic NF-κB signaling pathway (by targeting the anti-apoptotic protein 14-3-3ζ) [86,174,175]. Moreover, the tumor suppressor miR-218 regulates COX-2 (cyclooxygenase-2) via the anti-apoptotic NF-κB signaling pathway [155]. These findings suggest that the dysregulated miRs control mitochondria-mediated (intrinsic) and death receptor–mediated (extrinsic) apoptotic pathways through the Bcl-2 family target [216]. Further, dysregulated miRs are also involved in the anti-apoptotic PI3K/Akt and NF-κB signaling pathways which control apoptosis by Bad and the *XIAP* gene [217].

In summary, the abundance of miRs expression may accelerate cell cycle progression through direct or indirect regulation of CDK inhibitors and several cell cycle regulators. Moreover, the abundance of miRs expression also may influence anti-apoptosis or the pro-survival pathway by targeting apoptosis-associated proteins. They may play an important molecular role in GC progression.

### 2.2. GC-Related miRNAs in Cell Migration, Invasion, and Metastasis

Metastasis, a complex, multistep process that involves cytoskeleton remodeling, matrix metalloproteinases (MMPs), homing receptors and their ligands, intracellular signaling pathways (TGFβ and TGFβ/c-Met) and angiogenesis, is a hallmark of malignant tumors [218,219]. As noted above, Zhang *et al.* [68] identified RECK as a direct target of the oncomiR miR-21, and also found that oncomiR-21 is up-regulated in *Helicobacter pylori-*infected GC tissues. RECK might also possess anti-invasion, anti-metastasis and anti-angiogenesis functions through modulation of MMP2, MMP9 and MMP14 expression. In addition, miR-21 targeting of PDCD4 (programmed cell death 4) is associated with lymph node metastasis and venous invasion. Another report indicated that PTEN (phosphatase and tensin homologue) is a target of miR-21 that promotes anoikis through activation of the PI3K/Akt pathway [68,72]. In addition, Tsai *et al.* [59] have shown that oncomiR-196a/b expression promotes GC cell migration, invasion, and metastasis by increasing radixin (RDX) expression in GC tissues. OncomiR-370 was shown to decrease TGFβ-RII expression and stimulate TGFβ1-induced phosphorylation of Smad3. Thus, oncomiR-370 is capable of triggering cell migration by disturbing the TGFβ signaling pathway [85]. Moreover, oncomiR-215 was shown to target ALCAM (activated leukocyte cell adhesion molecule) and increase GC metastasis [71]. Conversely, the tumor suppressor miR-218 promotes invasion and metastasis by targeting Robo1, and thereby activating the Slit/Robo1 signaling pathway [156,157,158,159,160]. Other tumor suppressors of the let-7 family increase the expression of HMGA2 (high mobility group AT-hook 2), which is associated with tumor invasion and is an independent prognostic factor in GC [107]. Furthermore, members of the miR-200 family increase the epithelial-mesenchymal transition (EMT), and contribute to cell migration by reducing the expression of E-cadherin repressors ZEB1 and ZEB2 (zinc finger E-box binding homeobox 2) [145,146]. Down-regulation of miR-335 was found to be significantly associated with lymph node metastasis, invasion of lymphatic vessels, cell invasion and metastasis through targeting of BCL-w and SP1 (specificity protein 1) [82,83].

Interestingly, the function of miRNAs depends on the expression of their target genes. Previous studies revealed that some miRs could target both oncogenes and tumor-suppressor genes, leading to opposite roles in GC. Accordingly, miR-9 may play dual but opposing roles in GC. Thus, acting as an oncomiR, miR-9 targets CDX2 [102] and increases cell proliferation by facilitating cell cycle progression; conversely, acting as a tumor suppressor, miR-9 targets NF-κB1, cyclin D1, and ETS1 to contribute to anti-proliferation and anti-metastasis [102,112,113,114]. Nakayama *et al.* [20] reported that oncomiR-10b targets HOXD10 (homeobox D10) to promote GC metastasis. However, Kim *et al.* [116] found that miR-10b also represses the expression of MAPRE1 (microtubule associated protein RP/EB family member 1), resulting in the inhibition of colony formation and cell proliferation. Moreover, oncomiR-223 was shown to promote GC invasion and metastasis by targeting EPB41L3 (erythrocyte membrane protein band 4.1-like 3) expression [76]. However, Kang *et al.* [162,163] reported that miR-223 acted as a tumor suppressor, directly targeting STMN1 (stathmin 1) expression to inhibit cell growth and metastasis. Thus, some miRNAs play dual roles through targeting of different genes during GC progression. Further studies will be required to elucidate the details of these different roles.

## 3. Clinical Applications of MicroRNAs in GC

### 3.1. GC-Related miRNAs as Diagnostic Biomarkers

Early diagnosis permits effective and radical treatment of GC before it develops to an advanced and metastatic stage. Gastroscopy with biopsy, the current standard clinical practice, is not a good screen for GC on a population basis, and existing biomarkers exhibit poor sensitivity and specificity. Thus, there are currently no reliable diagnostic biomarkers for GC. Multiple or combined biomarker assays are expected to provide more accurate results [220]. Investigators continue their efforts to identify convenient, high-sensitivity, high-specificity, and noninvasive biomarkers for early GC diagnosis [185]. MiRNAs can be released from tumor tissues into bodily fluids, including serum, plasma, urine, tears, amniotic fluid and gastric juice, through the secretion of exosome particles [15,221,222]. Mitchell *et al.* [15,221,222] demonstrated that circulating miRNAs in plasma/serum from GC patients are consistent with those in tissues; therefore, they could be useful as noninvasive biomarkers for the initial diagnosis of GC and assessment of GC recurrence. The most widely investigated biomarkers have been discovered using newer methods, such as systematic analysis of miRNA profiling, miRNA profiling, microarray profiling, and Q-RT-PCR profiling approaches [223,224,225,226,227]. The major plasma/serum-based, GC-related circulating miRNAs that have been suggested as useful GC biomarkers are listed in Table 3 and Table 4.

Liu *et al.* [111] used systematic analysis of miRNA profiling, miRNA profiling to identify a signature of five circulating oncomiRs—miR-1, miR-20a, miR-27a, miR-34 and miR-423-5p—and correlated it with tumor stage. Using receiver-operating characteristic (ROC) curve analyses, these authors evaluated the diagnostic value of this miR signature, showing that it achieved a sensitivity of 80% and a specificity of 81%. They observed that the circulating five-oncomiR signature I exhibited a high diagnostic value, with an area under the ROC curve (AUC) of 0.879. By comparison, the five-oncomiR signature II exhibited an AUC of 0.831, which is higher than that of CEA, with an AUC of 0.503, and CA19-9, with an AUC of 0.6. In a large-scale analysis, four circulating oncomiRs (miR-17-5p, miR-21, miR-106a and miR-106b) significantly distinguished GC patients from healthy controls and pre-operative from post-operative GC patients [185]. Moreover, Valladares-Ayerbes *et al.* [42], using a Cox multivariate regression model, identified circulating oncomiR-200c as a biomarker for GC diagnosis and as an independent prognostic factor for progression-free survival and overall survival in GC patients. Liu *et al.* [49] found that oncomiR-378 in the GC patients was significantly higher than that in the healthy controls. OncomiR-378 exhibited a high diagnostic value, with an AUC of 0.861, a sensitivity of 87.5% and a specificity of 70.7%.

In addition, several oncomiRs circulating in the blood of GC patients can be used as diagnostic biomarkers to distinguish GC patients from healthy individuals. These include miR-1, miR-106a, miR-106b, miR-17, miR-17-5p, miR-18a, miR-192, miR-199a-3p, miR-20a, miR-200c, miR-21, miR-210, miR-218, miR-221, miR-222, miR-25, miR-27a, miR-34, miR-376c, miR-378, miR-421, miR-423-5P, miR-451, miR-486, miR-744, and miR-93 [26,27,54,68,71,80,111,184,185,186,187,188,190,191,195,196,197,199,200,203,206,207,208]. Of these, miR-17-5p, miR-18a, miR-20a, miR-200c, miR-21, miR-218, miR-221, miR-222, miR-25, miR-27a, miR-376c, and miR-744 were found to be significantly elevated in GC patients, and their expression was significantly reduced after surgery [26,27,54,68,71,80,81,155,187,192,193,195,196,198,199,200,201,202,203,204,205].

Conversely, several tumor-suppressor miRNAs circulating in the blood of GC patients can also be used as diagnostic biomarkers to distinguish GC patients from healthy individuals, including miR-122, miR-195-5p, miR-203, miR-218, and miR-375 [155,189,198,209,210,211]. Of these, miR-122, miR-203, and miR-218 were found to be significantly reduced in GC patients, and their expression was significantly increased following surgery [155,189,198,211].

Taken together, these findings suggest that circulating miRNAs are useful, noninvasive biomarkers for early diagnosis or monitoring of cancer survivors after treatment of GC. The significance of these biomarkers compares favorably to the use of the traditional biomarkers CEA or CA19.9 alone.

### 3.2. GC-Related miRNAs as Prognostic Biomarkers

To predict GC patient survival time, cancer progression (disease stage), prognostic outcome, lymph node metastasis or response to treatment is challenging. Recurrence is also a key problem leading to the failure of treatments, including radical or chemical treatment and surgical resection. Although the clinical outcome of GC has improved, prognostic indicators capable of predicting recurrence in GC patients after treatment are still lacking. Recently, due to the stability and specificity of expression in tissues and circulation, accumulating evidence has shown that miRNAs can be regarded as novel biomarkers with a potential clinical significance tool for GC patients’ outcomes.

In general, the occurrence of a distant metastasis frequently leads to advanced-stage cancer and shorter survival. In this context, it has been shown that oncomiR-10b, miR-21, and miR-212 in GC patients are associated with a high metastasis risk and poor clinical outcomes, including tumor-node-metastasis, tumor size, stage, lymph node metastasis, and five-year survival rate [69,126,228].

Li *et al.* [229] showed that a seven-miRNA signature (miR-10b, miR-21, miR-223, let-7a, miR-338, miR-30a-5p and miR-126) could predict relapse-free and overall survival of GC patients. In addition, oncomiR-20b, miR-150 [23], miR-214 [24,74], miR-375 [39,74,86,87,88], tumor suppressor Let-7g [24,109,110], miR-125-5p [126], miR-146a [24,134], miR-218 [154], miR-433 [24,86,109,110,174], and miR-451 [24,94,230] are associated with a poor survival prediction in GC. In GC, high expression of miR-195 [58], miR-199a [39,58,62,63,64,65], miR-1952 [58], miR-335 [82,83], miR-375 [39,74,86,87,88], miR-451 [58,94,95,96] and miR-4512 [39], and low expression of miR-142-5p [39] are more likely to indicate relapse or recurrence of GC patients. Moreover, GC patients with over-expression of miR-107 [28,29,30], miR-143 [40], miR-145 [41,42], miR-181b/c [17,47,48,55,56], miR-196a/b [59], miR-20b [23,66], miR-23a/b [77,78,79], miR-34 [17,47,48,55,56] and miR-630 [100] and decreased expression of miR-1 [111], miR-1207-5p [121], miR-125a-3p/-5p [24,125,126,127], miR-185 [140], miR-193b [60], miR-20a [111], miR-206 [150,151], miR-215 [142], miR-217 [153], miR-27a [111], miR-29c [169], miR-34a [172,173], miR-423-5p [111], and miR-520d-3p [99] indicate advanced tumor stage or TNM stage. High levels of miR-107 [28,29,30], miR-181b/c [17,47,48,55,56], miR-192 [57], miR-196a/b [59], miR-20b [23,66], miR-21 [68,69,70,71,72], miR-214 [24,74], miR-23a/b [77,78,79], miR-25 [26,27,80], miR-27a [23,81], miR-630 [100], and miR-650 [101] and decreased levels of Let-7g [24,109,110], miR-1207-5p [121], miR-125a-3p/-5p [24,125,126,127] and miR-126 [128,129,130], miR-146a [24,134], miR-148a [46,135,136,137], miR-153 [139], miR-218 [154,155,156,157,158,159,160], miR-22 [151,161], miR-27a [111], miR-29c [169], miR-335 [171], miR-34a [172,173], miR-429 [177], miR-433 [24,86,109,110,174] and miR-520d-3p [99] are associated with invasion or LNM, as well as metastasis.

Conversely, the tumor suppressors miR-125a and miR146a are significantly correlated with lymph node metastasis, indicating that they could be prognostic factors of overall survival [126,134]. Other study showed that low expression of let-7 is related to tumor invasiveness and prognosis by targeting HMGA2 [231].

Therefore, many potential predictors have been regarded as beneficial for mediating the prognosis of GC patients and are the basis for targeted therapy. In future, these prognostic miRNAs could be useful for making choices concerning treatment.

### 3.3. GC-Related miRNAs as Treatment Biomarkers

One miRNA may regulate multi-target gene expression and multiple pathways, affecting the process of tumor development [232,233,234]. Thus, miRNAs are more effective than coding genes as biological regulation molecules.

The methods of current miRNA-mediated treatment are focused on miRNA knockout or silencing the endogenous oncomiRs, including anti-miRNA oligonucleotides (AMOs) [13], miRNA sponges [235], miR-Mask [236], antagomiRs and miRNA inhibitors [12,237]. For example, Chun *et al.* [200] transfected AS-miR-221/222 with liposomes into GC cell line SGC7901 to inhibit GC cell growth and invasion. Moreover, high expression of miR-196a/-196b promotes GC cell migration and invasion. Elevated miR-196a/-196b expression results in decreasing target RDX protein in GC cells and *vice versa*. Similar results were obtained in a mouse model of human GC. Tsai *et al.* [59], through AMOs, used anti-miR-196a/-196b oligonucleotides or the over-expression of RDX, which may serve a therapeutic purpose to inhibit GC metastasis.

Conversely, forced expression the tumor suppressor miRNAs is used to gain the resolution of tumor treatment. miRNA over-expression is often performed by using an *in vivo* or *in vitro* RNA delivery system as in cancer therapeutics, including adeno-associated viruses [238] or nonpathogenic bacteria [239] as a carrier to introduce a specific miRNA or miRNA mimics to up-regulate miRNA [240]. miR-1207-5p and miR-1266 are significantly down-regulated in GC tissues. Over-expression of these two miRs inhibited GC growth through targeting hTERT system *in vitro* and *in vivo*. Chen *et al.* [241] showed a novel therapeutic method for the delivery of these two miRs for GC treatment.

However, some problems must be considered. For example, one miRNA modulates multi-target genes and multiple pathways. Also, the off-target effects are unexpected. Thus, better specificity and an effective miRNA delivery system for a therapeutic strategy must be developed [242,243].

## 4. Conclusions

miRNA biomarkers have been found at elevated levels in the blood or tissues of patients with tumors. Changes in different biomarkers during tumor progression can help clinicians monitor cancer status. Although higher levels of a biomarker can potentially predict a tumor, other factors may also account for such elevated levels. Dysregulation of tumor markers can occur in response to the presence of a tumor or a change in status, enabling them to be used for a range of applications, including screening, diagnosis, staging, prognosis, and monitoring of recurrence after treatment. The values of these miRNAs as biomarkers will require further confirmation in human GC patients. In the future, an miRNA or an miRNA signature could be a better diagnostic or therapeutic tool than a single gene. However, the challenge is to develop a standard protocol for collecting large specimens, re-analyzing them in a large independent cohort, and validating their significance in clinical applications.

Moreover, several studies have suggested that tumor-derived circulating miRs might be secreted into circulation. Circulating miRs in the plasma/serum of GC patients can be used as diagnostic biomarkers to compare GC patients with healthy controls [185,244]. Most of these biomarkers are more sensitive and specific than the traditional biomarkers CEA or CA19.9 alone [111,185,190]. These findings provide novel indicators for monitoring GC dynamics and early diagnosis for GC to improve survival.

Currently, biomarkers are only used as a reference and not to diagnose the disease *per se*. A professional physician will still need to provide a comprehensive judgment, including choice of tumor marker, evaluation of clinical symptoms, assessment of related imaging performance, and other non-specific factors. Ultimately, the personalized management, diagnosis, and prognosis of the disease can be achieved using a panel of miRNAs.

## Figures and Tables

**Table 1:** Up-regulated miRNAs in tissues for GC.

Tissue OncomiRs	Samples	Cell Functions	Target (Official Gene Name)	Clinical Application	References
**let-7b**	Systematic integrative bioinformatics framework	ND	ND	Diagnosis	[16]
**let-7g**	GCCLs	Chemosensitivity	ND	ND	[17]
**miR-10b**	GCCLs	Metastasis	*HOXD10*	ND	[18,19,20]
**miR-105**	GCTs	ND	ND	Diagnosis	[21]
**miR-106a**	GCCLs	Cell cycle	*RB1* *TIMP2* *FAS*	ND	[22]
**miR-106b-93-25 cluster**	GCTs GCCLs	Apoptosis Cell cycle	*BIM* *E2F1* *CDKN1A* *CDKN1B* *CDKN1C*	Diagnosis	[23,24,25,26,27]
**miR-107**	GCTs GCCLs	Invasion Metastasis	*CDK6* *DICER1*	LNM Tumor stage Prognosis	[28,29,30]
**miR-1271**	GCCLs	ND	*IGFIR* *MTOR* *BCL2*	ND	[31]
**miR-129**	GCTs GCCLs	Cell proliferation Cell cycle	*SOX2* *SOX4* *CDK6* *PDCD2*	Prognosis Diagnosis	[32,33,34,35]
**miR-130a**	GCCLs	Metastasis Invasion Cell proliferation	ND	ND	[36]
**miR-130b**	GCCLs	Apoptosis Epigenetic regulation Cell proliferation	*RUNX3* *BIM*	ND	[37]
**miR-135a**	GCTs	ND	ND	Prognosis	[38]
**miR-142-5p**	GCTs	ND	ND	Poor Survival Prognosis	[39]
**miR-143**	GCTs	ND	ND	Tumor stage Scirrhous type Prognosis	[40]
**miR-145**	GCTs GCCLs	Angiogenesis	*CDH2* *ETS1*	Tumor stage Scirrhous type Prognosis	[41,42]
**miR-146a**	GCCLs	Apoptosis Cell proliferation	*IRAK1* *TRAF6* *SMAD4*	ND	[43,44,45]
**miR-148a**	GCCLs	Invasion Metastasis Cell proliferation Cell cycle	*CDKN1B*	ND	[46]
**miR-150**	GCTs GCCLs	Apoptosis Cell proliferation	*EGR2*	Poor Survival Prognosis	[23]
**miR-15b**	GCCLs	Apoptosis	*BCL-2*	ND	[47,48,49]
**miR-155**	GCCLs	Apoptosis	*IKK-ε* *SMAD4* *FADD* *PLIα*	ND	[50,51,52,53]
**miR-16**	GCCLs	Chemosensitivity Apoptosis	*BCL-2*	ND	[17,47,48,49]
**miR-17**	GCCLs	Cell cycle	*CDKN1A* *UBE2C* *FBXO31*	ND	[54]
**miR-181**	GCCLs	ND	ND	ND	
**miR-181b/c**	GCTs GCCLs	Apoptosis Chemosensitivity	*NOTCH4* *K-RAS* *BCL-2*	Differentiation Invasive depth Tumor stage Prognosis	[17,47,48,55,56]
**miR-192**	GCTs	ND	ND	LNM Prognosis	[57]
**miR-195**	GCTs	ND	ND	Recurrence	[58]
**miR-196a**	GCTs GCCLs	Metastasis Invasion Migration	*RADIXIN*	Invasion depth Serosal invasion Lymphatic invasion LNM Distant metastasis TNM stage Peritoneal seeding Gross type Lauren subtype Prognosis	[59]
**miR-196a**	GCTs GCCLs	ND	ND	Differentiation	[60]
**miR-196a-5p**	GCTs	ND	ND	LNM TNM stage Prognosis	[61]
**miR-196b**	GCTs GCCLs	Metastasis Invasion Migration	*RADIXIN*	Invasion depth Serosal invasion Lymphatic invasion LNM Distant metastasis TNM stage Peritoneal seeding Gross type Prognosis	[59]
**miR-199a**	GCTs GCCLs	Cell proliferation Metastasis	*SMARCA2* *SMAD4* *MAP3K11* *ZHX1*	Recurrence Diagnosis Relapse	[39,58,62,63,64,65]
**miR-1952**	GCTs	ND	ND	Relapse	[58]
**miR-20a**	GCTs GCCLs	Cell cycle	*CDKN1A*	Diagnosis	[23,24,25]
**miR-20b**	GCTs	ND	ND	Poor Survival LNM Distance metastasis TNM stage Prognosis	[23,66]
**miR-200c**	GCTs GCCLs	Metastasis Chemoresistance	*E-CDH* *ZEB2* *RHO E*	ND	[67]
**miR-21**	GCTs GCCLs	Apoptosis Cell proliferation Invasion Cell cycle Metastasis Differentiation	*RECK* *PTEN* *SERPINI1* *PDCD4* *NF-KB* *CDKN1A* *E2F5* *CDKN1C*	LNM Prognosis	[68,69,70,71,72]
**miR-210**	Hp-positive human gastric biopsies/Hp-negative controls	ND	*STMN1* *DIMT1*	ND	[73]
**miR-211**	Systematic integrative bioinformatics framework	ND	ND	Diagnosis	[16]
**miR-213**	GCTs	ND	ND	Diagnosis	[21]
**miR-214**	GCTs	ND	ND	Poor Survival Invasion depth Lymph node metastasis Prognosis	[24,74]
**miR-215**	GCTs GCCLs	Metastasis	*ALCAM*	Prognosis	[71]
**miR-221/222**	GCTs GCCLs	Radioresistance Cell cycle	*CDKN1A* *CDKN1B* *CDKN1C*	Prognosis	[27,68]
**miR-2214**	GCTs	ND	ND	Advanced GC Prognosis	[75]
**miR-223**	GCCLs	Invasion Metastasis	*EPB41L3* *FBXW7* *HCDC4* *STMN1*	ND	[76]
**miR-23a/b**	GCTs GCCLs	Invasion Cell proliferation	*IL6R* *IRF1*	LNM TNM stage Prognosis	[77,78,79]
**miR-25**	GCTs GCCLs	Invasion Cell proliferation Migration	*CDKN1C* *BCL2L11* *FBXW7* *LASTS2* *RECK*	LNM Prognosis	[26,27,80]
**miR-27a**	GCTs GCCLs	Metastasis Cell proliferation	*APC* *PHB*	Lymph node metastasis Prognosis	[23,81]
**miR-335**	GCTs	Metastasis	ND	Recurrence Prognosis	[82,83]
**miR-34**	GCTs GCCLs	Chemosensitivity Apoptosis	*BCL-2*	Tumor stage Prognosis	[17,47,48,55,56]
**miR-342**	GCCLs	Chemosensitivity	ND	ND	[17]
**miR-362**	GCCLs	Apoptosis	*NF-KB*	ND	[84]
**miR-363**	GCCLs	Chemoresistance	ND	ND	[17]
**miR-370**	GCCLs	Metastasis	*TGF-β-RII*	ND	[85]
**miR-375**	GCTs GCCLs	Apoptosis Inhibits Helicobacter pylori-induced gastric carcinogenesis	*PDK1* *YWHAZ* *JAK2* *STAT3*	Poor Survival Relapse/Recurrence Prognosis	[39,74,86,87,88]
**miR-382**	GCTs GCCLs	Angiogenesis	*PTEN*	ND	[89]
**miR-421**	GCTs GCCLs	ND	*BAX* *BCL-2*	Diagnosis	[90,91]
**miR-43c**	GCTs GCCLs	Cell proliferation Cell cycle	*VEZT*	Epigenetic regulation Prognosis	[92]
**miR-442a**	GCCLs	Chemoresistance	ND	ND	[93]
**miR-451**	GCTs GCCLs	Apoptosis Radiosensitivity	*MIF*	Recurrence	[58,94,95,96]
**miR-4512**	GCTs	ND	ND	Relapse	[39]
**miR-4732-5p**	GCCLs	Chemoresistance	ND	ND	[93]
**miR-4758-3p**	GCCLs	Chemoresistance	ND	ND	[93]
**miR-503**	GCCLs	ND	*IGFIR* *BCL2*	ND	[97]
**miR-512-5p**	GCCLs	Apoptosis	*MCL-1*	ND	[98]
**miR-514b**	GCTs	ND	ND	Diagnosis	[21]
**miR-517**	GCCLs	Chemoresistance	ND	ND	[17]
**miR-518f**	GCCLs	Chemoresistance	ND	ND	[17]
**miR-519e**	GCCLs	Chemoresistance	ND	ND	[17]
**miR-520a**	GCCLs	Chemoresistance	ND	ND	[17]
**miR-520d/h**	GCCLs	Chemoresistance	*HDAC1*	ND	[17]
**miR-520d-3p**	GCTs GCCLs	Cell proliferation Migration Invasion	*EPHA2*	ND	[99]
**miR-548N**	GCTs	ND	ND	Diagnosis	[21]
**miR-630**	GCTs GCCLs	Invasion	ND	LNM Distant metastasis TNM stage Prognosis	[100]
**miR-650**	GCTs	ND	ND	Lymph node Metastasis Prognosis	[101]
**miR-708**	GCTs	ND	ND	Diagnosis	[16]
**miR-9**	GCCLs	Cell proliferation Cell cycle	*CDX2*	ND	[102]
**miR-92**	GCCLs	Cell proliferation Invasion	*FXR*	ND	[103]
**miR-92a**	GCTs	ND	*E2F1* *HIPK1*	Tumor growth Prognosis	[93,104]
**miR-93**	GCCLs	Apoptosis	*BIM* *DAB2*	ND	[23,24,25]

Chemoresistance drugs were cisplatin, 5-fluorouracil and hydroxy camptothecin. GCTs: Gastric cancer tissues, GCCLs: Gastric cancer cell lines, ND: not determined.

**Table 2:** Down-regulated miRNAs in tissues for GC.

Tissue Tumor Suppressor miRs	Samples	Cell Functions	Target (Official Gene Name)	Clinical Application	References
**Let-7a**	GCCLs	Cell proliferation Cell cycle Invasion	*RAB40C* *HMGA2* *CDC34* *CCR7*	ND	[105,106,107]
**Let-7f**	GCCLs	Metastasis	*MYH9*	ND	[108]
**Let-7g**	GCTs	ND	ND	Diagnosis Invasion depth Lymph node metastasis Poor Survival Chemoresistance Prognosis	[24,109,110]
**miR-1**	GCTs	ND	ND	Tumor stage Prognosis	[111]
**miR-9**	GCTs GCCLs	Cell proliferation Metastasis	*ETS1* *NFKB1* *CCND1* *CUL4A* *CDX2*	ND	[102,112,113,114]
**miR-10b**	GCTs GCCLs	Cell proliferation	*MAPRE1* *CCND1*	ND	[19,115,116]
**miR-101**	GCTs GCCLs	Metastasis	*EZH2* *COX2* *MCL1* *FOS*	ND	[117,118,119,120]
**miR-1207-5p**	GCTs GCCLs	ND	ND	LNM Lymphovascular invasion Stromal reaction type TNM stage Prognosis	[121]
**miR-124**	GCCLs	Cell proliferation Invasion	*ROCK1*	ND	[122]
**miR-124a**	GCCLs	Cell cycle	*CDK6*	ND	[123]
**miR-1246, miR-302a and miR-4448**	GCCLs	ND	*DYRK1A*	ND	[124]
**miR-125a-3p**	GCTs GCCLs	ND	ND	Invasion LNM Liver metastasis Tumor stage Tumor size Peritoneal dissemination Prognosis	[125]
**miR-125a-5p**	GCTs GCCLs	Cell proliferation Metastasis Invasion Migration	*ERBB2* *E2F3*	Invasion depth Liver metastasis Tumor stage Tumor size Poor Survival Prognosis	[24,126,127]
**miR-125-5p**	GCTs	ND	ND	Poor Survival Prognosis	[126]
**miR-126**	GCTs GCCLs	Cell cycle Cell proliferation Metastasis Invasion Migration	*CRK* *PI3KR2* *PLK2*	Lymph node metastasis Prognosis	[128,129,130]
**miR-126**	GCTs	ND	ND	Advanced GC	[128]
**miR-126**	GCCLs	Chemoresistance	ND	ND	[109]
**miR-129**	GCCLs	Proliferation Cell cycle	*CDK6*	ND	[131]
**miR-129-1-3p**	GCCLs	Migration	ND	ND	[34]
**miR-129-2**	GCTs GCCLs	Cell proliferation	*SOX4*	Epigenetic regulation Differentiation	[35]
**miR-141**	GCCLs	Invasion Cell proliferation Metastasis	ND	ND	[132]
**miR-142-5p**	GCTs	ND	ND	Relapse	[39]
**miR-143**	GCCLs	Cell proliferation	*AKT*	ND	[133]
**miR-145**	GCCLs	Cell proliferation	*IRS1*	ND	[133]
**miR-146a**	GCTs GCCLs	Invasion Migration	*EGFR* *IRAK1*	Lymph node metastasis Venous invasion Poor Survival Prognosis	[24,134]
**miR-148a**	GCTs GCCLs	ND	ND	Advanced GC	[135]
**miR-148a**	GCTs GCCLs	Metastasis	*DNMT1* *CDKN1B* *ROCK1*	Distant metastasis Organ invasion Peritoneal invasion Prognosis	[46,135,136,137]
**miR-148b**	GCTs GCCLs	Cell proliferation	*CCKBR*	ND	[138]
**miR-148**	GCTs GCCLs	ND	ND	Lymph node metastasis Prognosis	[135]
**miR-15b**	GCTs GCCLs	Chemoresistance	ND	ND	[47]
**miR-153**	GCTs GCCLs	Migration Invasion	ND	LNM Prognosis	[139]
**miR-155**	GCTs GCCLs	Cell proliferation Invasion Migration	*C-MYC*	ND	[130]
**miR-16**	GCTs GCCLs	Chemoresistance	*ND*	ND	[47]
**miR-181c**	GCTs GCCLs	Cell proliferation	*NOTCH4* *KRAS*	Transcriptional activation	[56]
**miR-185**	GCTs GCCLs	ND	ND	Prognosis TNM stage	[140]
**miR-19b**	GCTs GCCLs	ND	ND	Diagnosis	[104,141]
**miR-192**	GCTs GCCLs	ND	ND	Tumor sizes Borrmann type Prognosis	[142]
**miR-193b**	GCTs GCCLs	Invasion Metastasis	ND	Differentiation Lauren type Tumor stage Prognosis	[60]
**miR-196a**	GCTs GCCLs	Chemoresistance	ND	ND	[109]
**miR-20a**	GCTs GCCLs	ND	ND	Tumor stage Prognosis	[111]
**miR-200b**	GCTs GCCLs	Invasion metastasis	ND	ND	[143]
**miR-200 family**	GCTs GCCLs	EMT Chemoresistance Cell proliferation Invasion Migration Apoptosis	*ZEB1* *ZEB2* *CDH1* *BCL2* *XIAP*	ND	[109,144,145,146]
**miR-203**	GCTs GCCLs	Cell proliferation Invasion	*ABL1*	ND	[147,148]
**miR-204**	GCTs GCCLs	Cell proliferation Invasion	*EZR* *SOX4*	ND	[149]
**miR-206**	GCTs GCCLs	ND	*CCND2*	Venous invasion LNM Hematogenous recurrence PStage Prognosis	[150,151]
**miR-212**	GCTs GCCLs	Cell proliferation	*MECP2*	ND	[152]
**miR-215**	GCTs GCCLs	ND	ND	Borrmann type Tumor sizes pT stage Prognosis	[142]
**miR-217**	GCTs GCCLs	Differentiation Distant Metastasis Invasion	ND	Tumor size TNM stage Prognosis	[153]
**miR-218**	GCTs GCCLs	Metastasis Invasion	*ROBO1* *COX2* *NFkB* *ECOP* *VOPP1*	Lymph node metastasis Transcriptional activation Prognosis Advanced gastric cancer Prognosis	[154,155,156,157,158,159,160]
**miR-22**	GCTs GCCLs	ND	*SP1*	LNM Distant metastasis Tumor stage Prognosis	[151,161]
**miR-223**	GCTs GCCLs	Metastasis	*STMN1*	ND	[162,163]
**miR-24**	GCCLs	Cell cycle	*AE1*	ND	[164,165]
**miR-27a**	GCTs GCCLs	ND	ND	Tumor stage Lymph node metastasis TNM stag Prognosis	[111]
**miR-29a**	GCTs GCCLs	Cell proliferation Cell cycle Metastasis	*P42.3* *CDC42*	ND	[166,167,168]
**miR-29c**	GCTs GCCLs	ND	ND	Venous invasion TNM stage Prognosis	[169]
**miR-30b**	GCTs GCCLs	Apoptosis	*PAI-1*	ND	[170]
**miR-31**	GCTs GCCLs	Chemoresistance	ND	ND	[109]
**miR-335**	GCTs GCCLs	Metastasis Cell invasion	*BCL-W* *SP1*	Lymph node metastasis Prognosis Invasion of lymphatic vessels	[171]
**miR-338**	GCTs GCCLs	Chemoresistance	ND	ND	[109]
**miR-34a**	GCTs GCCLs	ND	*BCL2* *PDGFR* *YY1*	Lymph node involvement TNM stage Differentiation Recurrence Prognosis	[172,173]
**miR-34**	GCTs GCCLs	Cell proliferation	*BCL2* *NOTCH1* *HMGA2* *C-MYC* *SIRT1*	TNM stage Transcription Epigenetic regulation Prognosis	[111,172]
**miR-370**	GCTs GCCLs	ND	ND	Diagnosis	[31]
**miR-375**	GCTs GCCLs	Apoptosis Cell proliferation	*PDK1* *YWHAZ* *JAK2* *ERBB2* *STAT3* *TP53*	ND	[86,174,175]
**miR-410**	GCTs GCCLs	migration invasion	*MDM2*	ND	[176]
**miR-423-5p**	GCTs GCCLs	ND	ND	TNM stage Prognosis	[111]
**miR-429**	GCTs GCCLs	Cell proliferation Apoptosis	*C-MYC* *BCL2* *XIAP*	Lymph node metastasis Prognosis	[177]
**miR-433**	GCTs GCCLs	ND	*GRB2*	Diagnosis Invasion depth Lymph node metastasis Poor Survival Prognosis	[24,86,109,110,174]
**miR-449**	GCTs GCCLs	Cell proliferation Apoptosis Cell cycle	*GEMININ* *P42.3* *CCNE2* *GMNN* *MET* *CCNE3* *SIRT1* *CDK6*	ND	[131,166,178]
**miR-451**	GCTs GCCLs	Cell proliferation	*MIF*	Poor Survival Prognosis	[24,94]
**miR-486**	GCTs GCCLs	Cell proliferation	*OLFM4*	ND	[179,180]
**miR-512-5p**	GCTs GCCLs	Cell proliferation	*MCI-1*	ND	[98]
**miR-520d-3p**	GCTs GCCLs	ND	ND	Invasion depth LNM Tumor stage Prognosis	[99]
**miR-610**	GCTs GCCLs	Invasion Metastasis	ND	ND	[181]
**miR-7**	GCTs GCCLs	Invasion Metastasis Chemoresistance	ND	ND	[109,182]
**miR-9**	GCTs GCCLs	Cell proliferation Cell cycle	*RAB34* *CDX2* *NFKB1*	Diagnosis	[24,172,183]
**miR-98**	GCTs GCCLs	Chemoresistance	ND	ND	[109]

Chemoresistance drugs were cisplatin, 5-fluorouracil and hydroxy camptothecin. GCTs: Gastric cancer tissues, GCCLs: Gastric cancer cell lines, ND: not determined.

**Table 3:** Up-regulated circulating miRNAs for GC.

Circulating OncomiRs	Samples	Methods	Sensitivity	Specificity	AUC	Target (Official Gene Name)	Clinical Application	References
**miR-1**	164 GC 127 HC	Microarray + qRT-PCR	79.3	86.5	0.879	ND	Diagnosis	[111]
**miR-106a**	90 GC 27 HC	Microarray + qRT-PCR	48.2	90.2	0.684	ND	Diagnosis	[184]
**miR-106a**	69 GC 30 HC	Microarray + qRT-PCR	85.5	80	0.879	ND	Diagnosis	[185]
**miR-106b**	69 GC 30 HC	Microarray + qRT-PCR	ND	ND	0.72	ND	Diagnosis	[185]
**miR-106b**	40 Pre GC 20 Post GC	qRT-PCR	ND	ND	ND	ND	TNM stage Diagnosis Prognosis	[186]
**miR-17**	90 GC 27 HC	Microarray + qRT-PCR	48.2	90.2	0.743	ND	Diagnosis	[184]
**miR-17-5p**	79 Pre GC 30 Post GC 6 Relapse GC	qRT-PCR	ND	ND	ND	ND	Diagnosis Poor Survival Differentiation TNM stages Prognosis	[54]
**miR-18a**	82 GC 65 HC	qRT-PCR	ND	ND	ND	ND	Poor Survival LNM Pathological grade Prognosis	[54,187]
**miR-18a**	104 GC 65 HC	qRT-PCR	ND	ND	ND	ND	Diagnosis	[188]
**miR-192**	12 GC 12 HC	qRT-PCR	ND	ND	0.732	ND	Diagnosis Distant metastasis No Distant metastasis	[189]
**miR-199a-3p**	30 EGC 70 HC	Microarray + qRT-PCR	0.76	0.74	0.818	ND	Diagnosis	[190,191]
**miR-20a**	79 Pre GC 30 Post GC 6 Relapse GC	qRT-PCR	ND	ND	ND	ND	Poor Survival Differentiation TNM stages Prognosis	[54]
**miR-20a**	164 GC 127 HC	Microarray + qRT-PCR	79.3	86.5	0.879	ND	Diagnosis	[111]
**miR-200c**	67 GC 15 HC	qRT-PCR	65.4	100	0.715	*BCL2* *XIAP*	Diagnosis LNM Poor Survival Prognosis	[192,193]
**miR-21**	174 GC 39 HC	Microarray + qRT-PCR	56.7	94.9	0.81	ND	Diagnosis	[194]
**miR-21**	69 GC 42 Pre GC 42 Post GC	qRT-PCR	ND	ND	ND	*RECK* *PTEN* *SERPINI1*	Venous invasion Poor Survival Prognosis Differentiation LNM Poor Survival Prognosis	[68,71,195,196]
**miR-21**	103 GC 103 HC	qRT-PCR	ND	ND	ND	ND	Diagnosis Prognosis	[197]
**miR-218**	68 GC 56 HC	qRT-PCR	ND	ND	ND	*ECOP*	Metastasis Tumor stage Poor Survival Prognosis	[155,198]
**miR-221**	82 GC 46 Dysplasia 128 SG or CAG	qRT-PCR	ND	ND	ND	*CDKN1B* *CDKN1C* *PTEN*	Differentiation Poor Survival Prognosis	[27,199,200]
**miR-221**	82 GC 82 HC	qRT-PCR	82.4	58.8	ND	ND	Diagnosis	[199]
**miR-222**	114 GC 36 CAG 56 HC	qRT-PCR	66.1	88.3	0.85	*CDKN1B* *CDKN1C* *PTEN* *RECK*	Diagnosis LNM TNM stages Serosal Invasion Poor Survival Prognosis	[27,200,201,202]
**miR-25**	70 GC 70 HC	qRT-PCR	ND	ND	ND	*CDKN1C* *BCL2L11* *FBXW7*	LNM TNM stage Poor Survival Prognosis	[26,27,80,203]
**miR-25**	40 Pre GC 20 Post GC	qRT-PCR	ND	ND	ND	ND	TNM stage Diagnosis Prognosis	[186]
**miR-27a**	82 GC	qRT-PCR	ND	ND	ND	*PHB* *APC*	Metastasis Poor Survival Recurrent Prognosis	[81,204,205]
**miR-27a**	164 GC 127 HC	Microarray + qRT-PCR	79.3	86.5	0.879	ND	Diagnosis	[111]
**miR-34**	164 GC 127 HC	Microarray + qRT-PCR	79.3	86.5	0.879	ND	Diagnosis	[111]
**miR-376c**	82 GC 82 HC 46 dysplasia 128 SG or CAG	qRT-PCR	82.4	58.8	ND	ND	Diagnosis Differentiation Poor Survival Prognosis	[199]
**miR-378**	61 GC 61 HC	qRT-PCR	87.5	70.7	0.861	ND	Diagnosis	[206]
**miR-421**	90 GC 90 HC	qRT-PCR	ND	ND	ND	ND	Diagnosis	[207]
**miR-423-5P**	164 GC 127 HC	Microarray + qRT-PCR	79.3	86.5	0.879	ND	Diagnosis	[111]
**miR-451**	56 GC 30 HC	Microarray + qRT-PCR	96	100	0.96	ND	Diagnosis	[208]
**miR-486**	56 GC 30 HC	Microarray + qRT-PCR	86	97	0.92	ND	Diagnosis	[208]
**miR-744**	82 GC 82 HC 46 dysplasia 128 SG or CAG	qRT-PCR	82.4	58.8	ND	ND	Diagnosis Differentiation Poor Survival Prognosis	[199]
**miR-93**	40 Pre GC 20 Post GC	qRT-PCR	ND	ND	ND	ND	TNM stage Diagnosis Prognosis	[186]

AG: chronic atrophic gastritis; GC: Gastric cancer; HC: Healthy control; LNM: Lymph node metastasis; Pre: pre-operative; Post: post-operative; SG: superficial gastritis; qRT-PCR: Quantitative reverse transcriptase polymerase chain reaction; AUC: Area under curve; ND: not determined.

**Table 4:** Down-regulated circulating miRNAs for GC.

Circulating Tumor Suppressor miRs	Samples	Methods	Sensitivity	Specificity	AUC	Target (Official Gene Name)	Clinical Application	References
**miR-122**	12 GC 12 HC	qRT-PCR	ND	ND	0.808	ND	Distance metastases Poor Survival Prognosis No Distant metastasis Diagnosis	[189]
**miR-195-5p**	20 GC 190 HC	qRT-PCR	ND	ND	ND	ND	Prognosis	[209,210]
**miR-203**	154 GC 22 HC	qRT-PCR	ND	ND	ND	ND	Gender Lymphatic invasion Venous invasion Peritoneal metastasis Distance metastasis LNM Liver metastasis TNM stage Poor Survival Prognosis	[211]
**miR-218**	68 GC 56 HC	qRT-PCR	ND	ND	ND	*ECOP*	Metastasis Tumor stage Poor Survival Prognosis	[155,198]
**miR-375**	NA	Microarray + qRT-PCR	0.85	0.80	0.835	ND	Prognosis	[210]

GC: Gastric cancer; HC: Healthy control; Pre: pre-operative; Post: post-operative; qRT-PCR: Quantitative reverse transcriptase polymerase chain reaction; AUC: Area under curve; ND: not determined.

## Abbreviations

AE1: Anion exhanger-1; AFP: Alphafetoprotein; ALCAM: Activated leukocyte cell adhesion molecule; AMOs: Anti-miRNA oligonucleotides; AUCs: Areas under the ROC curves; CA19-9: Carbohydrate antigen 19-9; CCKBR: Cholecystokinin B receptor; CEA: Carcinoembryonic antigen; COX-2: Cyclooxygenase-2; Cyclin-CDK: Cyclin-dependent kinase; CDK6: Cyclin-dependent kinase 6; E2F1: E2F transcription factor 1; EGFR: Epidermal growth factor receptor; EGR2: Early growth response 2; EMT: Epithelial–mesenchymal transition; EPB41L3: Erythrocyte membrane protein band 4.1-like 3; ERBB2: Erb-b2 receptor tyrosine kinase 2; EZH2: Enhancer of zeste 2 polycomb repressive complex 2 subunit; FFPE: Formalin-fixed paraffin-embedded; FOS: FBJ osteosarcoma oncogene; GC: Gastric cancer; HMGA2: High mobility group AT-hook 2; HOXD10: Homeobox D10; IL6R: interleukin (IL)-6 receptor; MAPRE1: Microtubule associated protein RP/EB family member 1; MCL-1: Myeloid cell leukemia 1; MIF: Migration inhibitory factor; miRNAs: MicroRNAs; MMP: Matrix metalloproteinase; NF-κB: Nuclear factor-κB; OncomiRs: Oncogenic miRNAs; OS: Overall survival; p21: cyclin-dependent kinase inhibitor 1A; PDCD4: Programmed cell death 4; PFS: progression free survival; PI3K: phosphoinositide 3-kinase; Post-op: Post-operative; Pre-op: Pre-operative; PTEN: Phosphatase and tensin homologue; Q-RT-PCR: quantitative reverse transcription-polymerase chain reaction; RDX: Radixin; RECK: reversion-inducing cysteine-rich protein with kazal motifs; ROC: Receiver operating characteristic; RUNX3: Runt related transcription factor 3; SP1: Specificity protein 1; STMN1: Stathmin 1; TGF-β1: Transforming growth factor-β1; TNM: Tumor-node-metastasis; Tumor suppressor-miRs: Tumor suppressive miRNAs; ZEB2: Zinc finger E-box binding homeobox 2.
